# Stable *Agrobacterium*-Mediated Transformation of Maritime Pine Based on Kanamycin Selection

**DOI:** 10.1155/2013/681792

**Published:** 2013-11-24

**Authors:** José M. Alvarez, Ricardo J. Ordás

**Affiliations:** ^1^Laboratorio de Biotecnología Agroforestal, Escuela Politécnica de Mieres, Universidad de Oviedo, Calle Gonzalo Gutiérrez Quirós, 33600 Mieres, Spain; ^2^Department of Plant Biology and Forest Genetics, Uppsala BioCenter, Swedish University of Agricultural Sciences, P.O. Box 7080, 75007 Uppsala, Sweden; ^3^Área de Fisiología Vegetal, Departamento BOS, Universidad de Oviedo, Calle Catedrático Rodrigo Uría s/n, 33071 Oviedo, Spain

## Abstract

An efficient transformation protocol based on kanamycin selection was developed for *Agrobacterium*-mediated transformation of maritime pine embryonal masses. The binary vector pBINUbiGUSint, which contained *neomycin phosphotransferase II* (*nptII*) as a selectable marker gene and ***β**-glucuronidase* (*uidA*) as a reporter gene, was used for transformation studies. Different factors, such as embryogenic line, bacterial strain, bacterial concentration, and coculture duration, were examined and optimized. For selection of transformants, 15 mgL^−1^ kanamycin was used. The highest transformation efficiency (11.4 events per gram of fresh mass) was achieved when a vigorously growing embryonal mass (embryogenic line L01) was cocultivated with *Agrobacterium* strain AGL1 at the optical density (OD_600 nm_) of 0.3 for 72 h. Evidence of the stable transgene integration was obtained by polymerase chain reaction for the *nptII* and *uidA* genes and expression of the *uidA* gene. Maturation capacity of the transgenic lines was negatively affected by the transformation process. Induction of axillary shoots by preculturing the embryos with benzyladenine allowed overcoming the low maturation rates of some transformed lines. The transgenic embryos were germinated and the axillar shoots were rooted. Transgenic plants were transferred to potting substrate showing normal growth.

## 1. Introduction

Maritime pine (*Pinus pinaster* Ait.) is the most widely planted softwood species in France, Spain, and Portugal. It has also been widely cultivated in nonnative areas such as Australia, South Africa, South America, and New Zealand forming part of reforestation programmes. Biotechnological approaches for the improvement of maritime pine, such as *in vitro* propagation based on somatic embryogenesis (SE), offer new opportunities in the field of propagation and genetic engineering [1]. Genetic transformation of embryonal masses (EM) provides the potential to allow gene function analysis or to transfer specific traits into selected genotypes without affecting their desirable genetic background, and when associated with conventional breeding, it may provide a powerful tool for rapid increase in yield and wood quality. Transgenic studies aimed at shortening the juvenile phase, studying phytoremediation, altering the lignin biosynthesis pathway, and increasing cellulose accumulation have been carried out also in other tree species [2–4].

Somatic embryogenesis from immature zygotic embryos has been the most commonly used method for regeneration of transformed conifer plants. Embryonal masses provide a source of dividing cells that were recognized as the most competent cells for genetic transformation [5], and it is almost an unlimited source of starting material. Embryogenic cultures are amenable to *Agrobacterium*-mediated or biolistic transformation, and somatic embryos could be initiated from single cells [6]. Another advantage of SE is the possibility of cryopreserving the obtained transformed lines.

*Agrobacterium*-mediated transformation has become the preferred method since it has significant advantages over direct DNA delivery (e.g., biolistic), such as more predictable transgene integration patterns, and the introduction of one or a few copies of genes into the plant genome decreasing the probability of gene silencing [7], high coexpression of introduced genes, less fragmentation of the transgene [8]. In addition, *Agrobacterium* was a much more efficient transformation tool in compatible plant species compared with the biolistic protocol [9]. The *Agrobacterium*-mediated method has been widely applied to different tissues of several pine species. However, it has been shown that transformation efficiency was strongly affected by EM genotype and age, the *A. tumefaciens* strain, the cocultivation protocol, control of bacterial growth with bactericides, and selection procedure [5].

An efficient transformation procedure is a prerequisite for functional genomic studies, such as studying metabolic pathways or validation of candidate genes. Most of these studies regarding conifers are being carried out in *Arabidopsis*. However, the differences between two evolutionary distant genera, *Arabidopsis* (angiosperms) and *Pinus *(gymnosperms), call for a specific analysis in the latter [10]. Thus, additional research is needed to further refine *Agrobacterium*-mediated protocols in order to broaden the range of transformable genotypes, selective agents, or *Agrobacterium* strains. The selective genes involved in stable transformation studies are (1) *neomycin phosphotransferase II* (*nptII*) that confers resistance to kanamycin, (2) *hygromycin phosphotransferase *(*hpt*) that confers resistance to hygromycin, and (3) the herbicide-resistant gene *bar* that confers resistance to phosphinothricin. Embryonal masses from *P. pinaster* were previously used for genetic transformation of French [11] and Portuguese [12] genotypes. The two reports described hygromycin selection, but transgenic plants were obtained only in French genotypes. More recently, Trontin et al. [5] mentioned the use of herbicide resistance selection with similar or higher transformation efficiencies than hygromycin. Although kanamycin is the most widely used antibiotic for plant transformation, few reports about the successful use of kanamycin in maritime pine have been made [10, 13]. In conifers, kanamycin selection seems to be a good choice for zygotic embryos of *Picea glauca* [14], *Larix kaempferi* X *L. decidua* [15], *Pinus taeda* [16], and *Pinus strobus* [17] and somatic embryos of *Picea abies* [18], *Pinus strobus* [19, 20], and *Pinus radiata* [21]. However, it was problematic in cotyledons of* Pinus radiata *[22], *P. pinea* [23], and *P nigra* [24]. The sensitivity of a particular tissue to kanamycin is a key element in the development of any new transformation system in which a kanamycin resistance gene is used [23]. 

The objective of the present study was to develop a transformation protocol for *Pinus pinaster* EM based on kanamycin selection of transformation events allowing the direct use of binary plasmids harboring the *nptII *gene developed for the study of gene expression. In this work, the genetic transformation of 5 maritime pine embryogenic lines (Spanish genotypes) through cocultivation with *A. tumefaciens* was studied and the sensitivity to kanamycin is presented and compared with hygromycin sensitivity. The analysis of several factors such as the *A. tumefaciens* strain, bacterial concentration, and duration of coculture has improved the transformation efficiency of this species. In our laboratory, the protocol presented in this study is being successfully applied to produce transgenic plants and to study genetic regulation in conifers [10, 13]. In addition, axillary shoots were induced by benzyladenine [25] in the transgenic embryos to overcome the low maturation rates of some transformed lines.

## 2. Materials and Methods

### 2.1. Plant Material and Culture Conditions

Embryogenic cultures of maritime pine were initiated from Spanish trees located in Asturias in 2009. Immature zygotic embryos were treated according to Lelu-Walter et al. [26] with some modifications; Westvaco WV5 medium [27] supplemented with 1 gL^−1^ casein hydrolysate, 0.5 gL^−1^ L-glutamine, 30 gL^−1^ sucrose, 4.4 *μ*M benzyladenine (BA), 9 *μ*M 2,4-dichlorophenoxyacetic acid (2,4-D), 4 gL^−1^ Gelrite (all purchased from Duchefa, Haarlem, The Netherlands), and pH 5.7 was used for initiation. Proliferating EM were subcultured on the same maintenance medium with the concentration of plant growth regulators (PGR) reduced to one-half. Embryogenic lines obtained were cryopreserved according to Alvarez et al. [28] when they were 3 months old. Five lines (L01, L05, L13, L15, and L26) characterized by high somatic embryo maturation yields were recovered from cryopreserved stock and used for transformation experiments.

### 2.2. Embryogenic Line Sensitivity to Selective Agents

The selective agents kanamycin and hygromycin (Duchefa) were tested. Five mL of a fine suspension of EM from each line assayed (100 mg mL^−1^) was poured onto a filter paper disc (Whatman number 2, 7 mm diameter) and drained by a low-pressure pulse in a Buchner funnel. The filter paper disc was placed on maintenance medium (without casein hydrolysate) supplemented with 500 mgL^−1^ cefotaxime (bactericide agent; control) or with 500 mgL^−1^ cefotaxime plus the selective agent at 1, 5, 10, 15, 20, or 25 mgL^−1^. The filter paper discs were subcultured onto fresh medium every 2 weeks for 3 subcultures. The relative fresh weight increment was calculated at the end of the treatment.

### 2.3. *Agrobacterium* Strains and Transformation Protocol

Three disarmed *Agrobacterium tumefaciens* strains were used in the transformation experiments: EHA105 [29], LBA4404 [30], and AGL1 [31]. These strains harbored the binary vector pBINUbiGUSint (see Resource 1 in Supplementary Material available online at http://dx.doi.org/10.1155/2013/681792, [32]) developed in our laboratory, a derivative of pBIN19 [33], carrying the *neomycin phosphotransferase II* (*nptII*) and the **β*-glucuronidase* (*uidA*; *GUS*) genes driven by the nopaline synthase promoter (NOS-P) and the promoter of the *ubi1* gene of maize polyubiquitin [34], respectively. Both genes carried the NOS-pA terminator. The *uidA* gene used in these experiments contained the PIV2 intron of the *ST-L1* gene from *Solanum tuberosum* within its coding sequence (*uidA-int*), thereby preventing its expression in *Agrobacterium* [35]. Bacteria at optical densities (OD_600 nm_) of 0.3, 0.15, and 0.075 were cocultured with the EM for 48 and 72 h.

The inoculation and cocultivation procedure was based on the protocol of Levée et al. [19] with some modifications. Bacterial cultures were started from glycerol stocks on solid YEP medium [36] containing 50 mgL^−1^ rifampicin (chromosomal selection) and 50 mgL^−1^ kanamycin (pBINUbiGUSint selection) for 48 h at 27°C. One colony was grown in 2 mL liquid YEP medium with 50 mgL^−1^ kanamycin overnight at 27°C and 150 rpm and then diluted 1 : 100 in the same medium and grown for 16 h. The suspension was centrifuged at 4,000 rpm for 10 min and the pellet resuspended in liquid WV5 medium to the desired optical densities (OD_600 nm_). Then, the necessary amount of EM to obtain a suspension of 100 mg mL^−1^ was added and disaggregated by vortex pulses. Acetosyringone (100 *μ*M) was added according to López et al. [24]. The suspension was poured onto filter paper (5 mL per 7 cm diameter filter disc) and drained using a low-pressure pulse in a Buchner funnel. The filter discs were placed on cocultivation medium (maintenance medium without casein hydrolysate) in a 90 mm diameter × 14 mm depth Petri dish sealed with paraffin film (Parafilm), in darkness at 23°C for 48 or 72 h. After cocultivation, paper discs were washed four times with 10 mL WV5 liquid medium in a Buchner funnel, drained with low-pressure pulse, and placed on decontamination medium (cocultivation medium with 500 mgL^−1^ cefotaxime). After 1 week, the paper discs were transferred onto selective medium (decontamination medium with 15 mgL^−1^ kanamycin) and subcultured every 2 weeks. Each embryogenic event growing on selective medium was considered as a putative independent transformation event and was isolated and grown on selective medium for at least six more subcultures.

The number of transformation events per gram of fresh mass for each *Agrobacterium* strain was recorded after 120 days for the embryogenic line L01. Ten independent putative kanamycin-resistant events from line L01 were selected for molecular and histochemical assays. These lines were cryopreserved and subjected to maturation and germination.

### 2.4. Maturation and Conversion into Plants

Maturation and germination were based on Alvarez et al. [25]. For maturation, 150 mg EM from each transformed line was disaggregated in 2 mL sterile water. The suspension was poured onto a piece of autoclaved filter paper disc, drained using a low-pressure pulse in a Buchner funnel, and placed on WV5 medium supplemented with 550 mgL^−1^ L-glutamine (Duchefa), 525 mgL^−1^ L-asparagine (Duchefa), 175 mgL^−1^ L-arginine (Duchefa), 19.75 mgL^−1^ L-citrulline (Sigma), 19 mgL^−1^ L-ornithine (Duchefa), 13.75 mgL^−1^ L-lysine (Duchefa), 10 mgL^−1^ L-alanine (Duchefa), 8.75 mgL^−1^ L-proline (Duchefa), 60 gL^−1^ sucrose, and 9 gL^−1^ Gelrite. After 1 week, the paper disc was transferred onto the same medium supplemented with 80 *μ*M abscisic acid (ABA) (Duchefa). Amino acids and ABA were filter-sterilized and added after autoclaving. Cultures were subcultured on fresh medium every 3 weeks for 3 months.

Mature somatic embryos of each transformed line were isolated and placed on PGR-free WV5 medium supplemented with 30 gL^−1^ sucrose and 4 gL^−1^ Gelrite until radicle elongation and bud breaking were evident. In order to overcome the problems associated with low maturation rates in some transformed lines, axillary shoots were induced in mature somatic embryos by preculturing with BA (10 *μ*M) for 7 days before transferring to germination medium [25]. Axillary shoots were isolated and rooted according to Álvarez et al. [37]. Then, plants from germinated embryos and rooted shoots were placed in a peat-vermiculite substrate (1 : 1 v/v) and grown at 95% relative humidity (RH) controlled by a fog system. RH was reduced by 5% every 3 days. After approximately 3 weeks, the plants were transferred to ambient humidity conditions in the greenhouse. 

### 2.5. Molecular Analysis

Molecular analysis was performed on 10 independent kanamycin-resistant embryogenic lines. The putative transgenic events were PCR-tested for the *nptII*, *uidA*, and *virG* genes. A noninoculated line and the AGL1 strain carrying the pBINUbiGUSint plasmid were used as negative and positive controls, respectively. Genomic DNA was extracted using the NucleoSpin Plant II Kit (Macherey-Nagel, Germany). The amplification was performed in a Biometra T-Gradient Thermoblock thermocycler with the Kapa Taq PCR kit (Kapa Biosystems Inc., Woburn, MA, USA). Approximately 10 ng template was amplified in 10 *μ*L reactions using the following PCR protocol: 95°C 5 min; 35 cycles of 95°C 30 s, 60°C 30 s, and 72°C 1 min; 72°C 5 min. Primers used are listed in Online Resource 2. Five transgenic plants (one-year-old) from germinated embryos and five rooted shoots were also PCR-tested for the *nptII*,* uidA*, and *virG* genes as above.

Transgene copy number was estimated by real-time PCR [38] using the comparative Ct method [39]. The analysis was performed on an ABI PRISM 7900HT instrument (Applied Biosystems Inc.) using the Fast SYBR Green Master Mix (Applied Biosystems Inc.). Reaction efficiency and Ct were calculated using the LinRegPCR software [40]. Approximately 10 ng DNA was amplified per 10 *μ*L reaction using the following protocol: 95°C 20 s, 45 cycles of 95°C 1 s, and 60°C 20 s. The *Pips-C61* gene (GenBank AJ490522), reported as a single-copy gene in the *P. pinaster* genome [41], was selected as endogenous control. Real-time PCR specificity was assessed using negative controls (no template), a melting curve analysis, and by gel electrophoresis. Three biological and two technical replicates were used per analysis. Primers (listed in Online Resource 2) were designed to amplify a fragment of the *uidA* (GUS) and *Pips-C61* genes, both with a 60°C Tm. The transgenic line T1 showed the lowest ΔCt, ΔCt being the difference between Ct for transgene and Ct for endogenous control (Ct_GUS_ − Ct_Pips-C61_), and was set as calibrator. The copy number was calculated as *E* ^−ΔΔCt^, where *E* = PCR efficiency and ΔΔCt = ΔCt sample – ΔCt calibrator. The difference (ΔCt) between the transgene Ct and endogenous control Ct was constant, independent of the amount of chromosomal DNA when PCR efficiencies of endogenous control and transgene were the same [38]. The transgenic T1 line was confirmed as harboring a single copy by Southern blot analysis (not shown).

### 2.6. *β-Glucuronidase* Assay during Embryo Development

*β-Glucuronidase* (GUS) activity was analysed fluorometrically and histochemically, both according to Jefferson et al. [42]. The assays were carried out on 10 kanamycin-resistant independent embryogenic lines. The fluorometric assay was performed on a TKO 100 Fluorometer (Hoefer Inc., MA, USA). Approximately 100 mg proliferating EM from each line were used. GUS activity is expressed as picomoles of methylumbelliferone (MU) per minute and per milligram of total protein. Total protein was quantified by the Bradford method [43]. Three independent assays were performed and samples were analysed in triplicate. The histochemical GUS assay was performed 15, 45, and 90 days after transfer onto maturation medium and in young needles from the transgenic plants. Blue colour development was evaluated after 16 h incubation at 37°C in GUS solution. 

### 2.7. Data Analysis

For kanamycin sensitivity tests, the samples were weighed at day 0 and after three subcultures. Relative fresh weight increment was calculated as follows: ΔFW_3_ = (FW_3_ − FW_0_)/FW_0_. A ΔFW_3_ < 2 was considered as growth inhibition.

An evaluation of maturation was determined by counting the number of mature somatic embryos per gram fresh tissue after 3 months on maturation medium. A mature somatic embryo was a white to yellowish embryo on which cotyledons are visible. This corresponds to stage 3 of somatic embryo development in *P. pinaster* as defined by Ramarosandratana et al. [44]. 

Data are presented as mean ± standard error of three independent replicates per experiment. Each experiment was performed at least twice at different times and assayed using a completely randomized design. The statistical analysis of categorical variables was carried out with the *χ* ^2^ test for overall and pairwise comparisons, except where indicated. Quantitative data were analyzed by analysis of variance (ANOVA) using the Holm-Sidak test for *post hoc* comparisons. Differences were considered significant at the 5 percent level. All statistical tests were performed with SigmaPlot v11.0 (Systat Software, Inc., San Jose, CA, USA).

## 3. Results

### 3.1. Embryogenic Line Sensitivity to the Selective Agents

**Figure 1:**
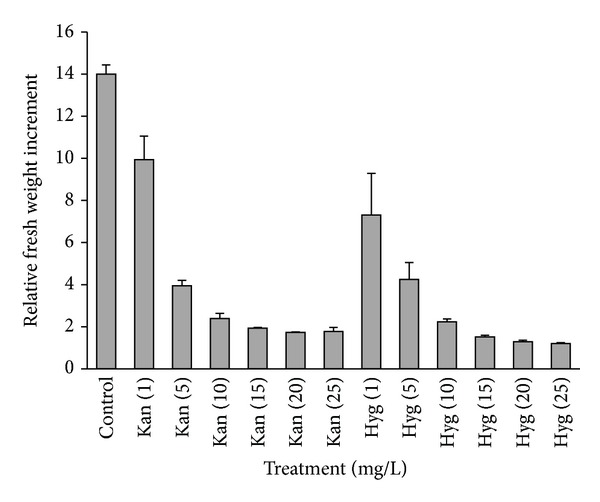
EM sensitivity to the selective agents. Kan: kanamycin, Hyg: hygromycin. Data were collected after 3 subcultures (6 weeks) and the values for the 5 embryogenic lines were averaged.

To determine the sensitivity of embryogenic cultures to the selective agents, untransformed lines (L01, L05, L13, L15, and L26) were cultured on maintenance medium supplemented with 500 mgL^−1^ cefotaxime plus the selective agent at different concentrations. The selective agents tested inhibited the growth of the EM. Kanamycin and hygromycin showed a similar behaviour since a concentration of 15 mgL^−1^ was enough to inhibit growth in all lines tested (Figure 1), and after three subcultures, the tissue necrotized (Online Resource 3).

### 3.2. *Agrobacterium* Strains and Transformation Protocol

The susceptibility of maritime pine embryogenic cultures to infection with three different *A. tumefaciens* strains (EHA105, LBA4404, and AGL1) harboring the pBINUbiGUSint plasmid was also studied. Cefotaxime was successfully used to suppress the growth of the three *Agrobacterium* strains tested. No transformation events were obtained for lines L13, L15, and L26. Line L05 showed transformation events only with the AGL1 strain at OD_600 nm_ 0.3 and a 48 h cocultivation (6.3 ± 0.7 transformation events per gram of fresh mass). Line L01 showed transformation events with AGL1 and EHA105 strains, but none were obtained with the LBA4404 strain. After 120 days of culture, the number of transformation events per gram of fresh mass varied significantly, depending on the strain used (Table 1). 

About 2 months after transformation, white proliferating masses (Online Resource 4) were detected, and these were subsequently transferred to fresh selective medium. After a further 3 months of culture on selective medium, the proliferating EM were considered to be putatively transgenic clones and were selected for molecular and histochemical analysis.

### 3.3. Maturation and Conversion into Plants

**Figure 2:**
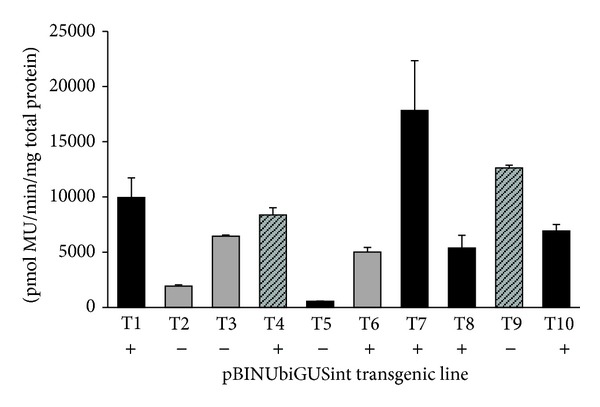
Fluorometric assay of 10 kanamycin-resistant lines. Different bar fills indicate transgene copy number: 1 copy (solid black), 2 copies (striped grey), and 3 or more copies (solid grey). The presence (+) or absence (−) of maturation is depicted under each transgenic line.

For maturation, kanamycin and cefotaxime were removed. No *Agrobacterium* regrowth was observed. Mature embryos were obtained from 6 out of 10 transgenic lines (Figure 2). The number of mature embryos showed a high variability among lines, on average with 13.8 ± 3.4 mature embryos per gram of fresh mass. This value was significantly lower than 231.9 ± 7.1 mature embryos per gram of fresh mass in the untransformed line L01 at the same age. No correlations were observed among maturation capacity, number of T-DNA insertions, and GUS activity (Figure 2). Transgenic lines that showed mature embryos were cryopreserved and recovered. No reduction in maturation capacity was observed after cryopreservation.

After 1 month on germination medium, 68.2% of embryos showed radicle elongation and bud-break. These were transferred to peat-vermiculite substrate and plant conversion was 71.4 %. 

No significant differences on germination or acclimatization percentages were observed when the transgenic embryos were treated with BA before transferring to germination medium compared with nontreated embryos. In addition, various axillary shoots per plantlet (5-6) were obtained after 3 months of culture on germination medium. The lateral shoots were isolated and rooted (85%). No plagiotropic growth was observed and the plants showed a well-developed root system capable of sustaining further shoot outgrowth. 

### 3.4. Molecular Analysis

Ten putative transgenic lines resistant to kanamycin were tested by PCR to detect the *nptII* and *uidA *genes (included in the T-DNA) and the* virG* gene (to detect bacterial contamination). All of the lines were PCR-positive for both *nptII* and* uidA *genes, so no escapes were detected. The *virG* gene was only amplified in the positive control (AGL1 pBINUbiGUSint). No amplification was detected in the negative control (nontransformed L01 line) (Online Resource 5). The five transgenic plants (one-year-old) from germinated embryos and the five rooted shoots tested for the *nptII *and *uidA* genes were PCR-positive. No amplification was detected for the *virG* gene (Online Resource 6). Copy number estimation by the comparative Ct method showed one copy in five lines, two copies in two lines and three or more copies in three lines (Figure 2). 

### 3.5. *β-Glucuronidase* Assay during Embryo Development

**Figure 3:**
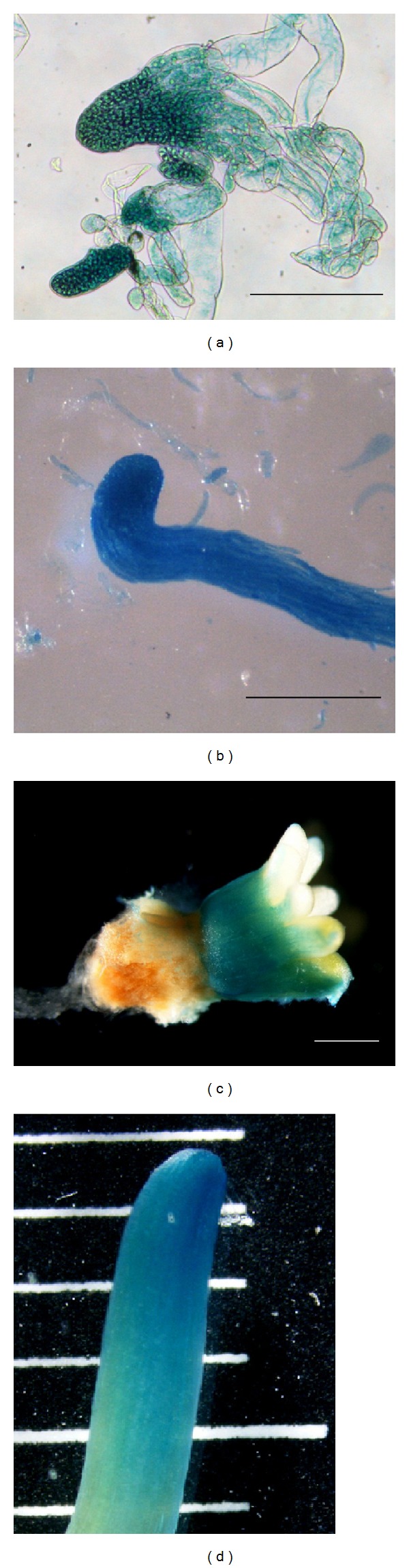
GUS activity after 16 h incubation in GUS solution. Embryos in different stages after 15 (stage 1 embryo; (a)), 45 (stage 2 embryo; (b)), and 90 days (stage 3 embryo; (c)) on maturation medium. Young needle (d). Bar and divisions = 1 mm.

The presence of GUS activity in the EM and embryos harboring the *uidA* gene was investigated by histochemical assay. All transgenic lines were positive during EM proliferation and maturation. GUS staining in mature embryos (stage 3) was often located in the hypocotyl. Young needles also showed GUS staining. No chimeric tissue or escapes were observed (Figure 3).

The GUS fluorometric assay revealed significant differences in activity levels (from 550.0 ± 22.1 to 17,831.2 ± 4,501.3 pmol MU min^−1^ mg^−1^ of total protein) for the 10 transgenic lines during EM proliferation (Figure 2). No significant correlations were found between GUS activity levels and transgene copy number. Both the highest and the lowest expression levels were found in single-copy lines.

## 4. Discussion

Somatic embryogenesis in *P. pinaster* has been improved in recent years [1, 26, 45] providing a source of competent cells for genetic transformation. Genetic engineering can facilitate the introduction of economically important genes that may otherwise be difficult to integrate into elite genotypes [5], especially in forest tree species with long reproductive periods where conventional breeding can pose a long-term challenge. Furthermore, genetic transformation is a very attractive alternative for studying candidate gene function. To produce stably transformed plants, the desired DNA has to be introduced into plant cells and integrated into the cell genome. These transgenic cells must then be selected, multiplied, and finally regenerated into a plant. Therefore, development of efficient gene delivery systems based on efficient *in vitro* plant regeneration protocols is a prerequisite for the application of genetic transformation in any species. 

In this work, we report obtaining transformed plantlets from *P. pinaster* EM based on kanamycin selection. Various factors influencing the efficiency of T-DNA delivery into maritime pine embryogenic cells via *A. tumefaciens *were evaluated. Cefotaxime, a decontamination agent used to inhibit *Agrobacterium* growth following infection [23], was successfully used to suppress the growth of the three *Agrobacterium *strains tested. Explants showed vigorous growth after 6 weeks of culture in the presence of cefotaxime (Online resource 3). AGL1 strain was confirmed as the superior tested strain and was efficiently used, thereby broadening the range of *Agrobacterium* strains for maritime pine transformation. AGL1 is a disarmed derivative of C58, a hypervirulent strain that has been successfully used to infect various plant species [46, 47]. However, this strain has been rarely employed in pine. Trontin et al. [5] reported very low transformation frequencies with AGL1 as compared with LBA4404 and C58pMP90 strains in a French genotype. This suggests a genotype-dependent compatibility. We have achieved genetic transformation in 2 out of the 5 Spanish genotypes tested, which demonstrates the known importance of genetic background on transformation efficiency [5] and stresses the need of further research to increase the range of transformable genotypes. We obtained a transformation efficiency of 11.4 events per gram of fresh mass. This result is in the range obtained in previous reports working with hygromycin resitance in French genotypes (0.03 to 88.29 events per gram of fresh mass [11]) or Portuguese genotypes (0.00 to 24.67 events per gram of fresh [12]). 

pBINUbiGUSint, the kanamycin resistance-harboring binary vector used in this work, has been proven successful in woody species, such as *Castanea sativa* [48], *Quercus suber* [46], and *Olea europaea* [47] and in *Pinus* spp. transient expression studies in our laboratory [24, 32, 49]. Although kanamycin has been shown to allow morphogenesis in several species and certain species were quite resistant to kanamycin, others such as *Pinus pinea* and *P. radiata* [22, 23] were extremely sensitive, suggesting that other selection strategies might produce better results. However, our data (Figure 1) showed a similar sensitivity of maritime pine EM to kanamycin and hygromycin. 

Constructions harboring hygromycin resistance have been successfully used in maritime pine EM transformation by other authors [11, 12]. However, obtaining transformed plantlets has been reported only once [11]. More recently, Trontin et al. [5], referring to unpublished data, suggested the use of phosphinothricin-based methods as a better alternative than hygromycin to select transgenic lines in maritime pine. Our results suggest that kanamycin selection is also a suitable alternative. 

The proliferation rate of inoculated cultures was affected by the bacterial strains tested at the beginning of the culture. Although there was no early bacterial regrowth after cocultivation, the infection reduced the proliferation rate of maritime pine EM (data not shown). Delaying the transfer of the cultures to the selective medium for 7 days [19] has allowed the embryogenic cultures to recover from the inoculation stress, proliferate, and accumulate the selectable enzyme.

Maturation of transformed lines in pine has proved to be difficult [12, 50]. A significant reduction in maturation capacity from 231.9 ± 7.1 in the untransformed line to an average of 13.8 ± 3.4 stage 3 embryos per gram of fresh mass in the transgenic lines was found. Tereso et al. [12] reported a strong reduction in maturation capacity of transformed lines since only 3 mature embryos were obtained from 44 lines selected with hygromycin. Trontin et al. [5] also reported a reduction in maturation yield of transformed lines using both hygromycin and phosphinothricin-based methods in the embryogenic line PN519. This loss of maturation capacity could be related to the transformation process itself, which could accelerate the loss of maturation capacity in the EM, or the mutagenic effect of T-DNA insertions [51] but further experiments are necessary for confirmation. Our results support the induction of axillary shoots by preculturing somatic embryos in the presence of BA [25] as a useful method to amplify and propagate low-maturating transgenic lines.

Expression (measured as GUS activity) of *uidA* gene was detected in all transformed lines of *P. pinaster* during somatic embryogenic mass proliferation and somatic embryo maturation; control cultures did not show any detectable GUS expression. Transgenic cultures proliferated in selective medium and expressed the *uidA* gene carrying the PIV2 intron, thereby demonstrating its functionality. High variability in GUS activity was observed between the different transformed clones. A comparison between GUS activity and transgene copy number suggests that the different level of gene expression cannot be explained by the copy number effect [52, 53]. Therefore, other phenomena such as the position effect in the host genome [54] or other complex configurations of the integrated T-DNA [7, 55] should also be considered. 

GUS staining was observed in all embryogenic phases, from proembryogenic masses to mature embryos and young needles. No escapes or chimeras were observed in the transgenic lines. These results support kanamycin selection as a suitable alternative for maritime pine genetic transformation.

In conclusion, a transformation method based on kanamycin selection broadens the range of selective agents reported for maritime pine EM transformation, and two new genotypes susceptible to *Agrobacterium*-mediated transformation are presented. The protocol is being successfully applied to produce transgenic plants and to study genetic regulation in conifers in our laboratory [10, 13]. In previous experiments, we observed that maritime pine embryogenic cultures were susceptible to transformation with AGL1 harboring a binary vector carrying the *PipsRR1* (GenBank JQ801609) promoter driving GFP:GUS expression [13]. That study was the first report on the development of a protocol to transfer foreign chimeric genes under a maritime pine promoter and one of the few reports on pines. We have also analysed the chimeric gene under the control of the *PipsCLV1L *(GenBank HQ377527) promoter [10]. These studies showed that an efficient transformation procedure was a prerequisite for comparative functional genomic studies between two evolutionary distant genera, such as *Arabidopsis* (angiosperms) and *Pinus* (gymnosperms).

## Supplementary Material

Supplementary Data: Supplementary Data are available online. Online Resource 1: T-DNA structure of the binary vector pBINUbiGUSint. Online Resource 2: Primer sequences used for PCR amplification and Real Time PCR (qPCR). Online Resource 3: Appearance of the EM after 3 subcultures (6 weeks) on selective medium. Online Resource 4: Putatively transformed masses. Online Resource 5: PCR amplification of the genes nptII, uidA, and virG in 10 putative transgenic lines. Online Resource 6: PCR amplification of the genes nptII, uidA, and virG in 5 transgenic plants and 5 rooted shoots.

## Figures and Tables

**Table 1:** Number of transformation events per gram of fresh mass in line L01 after 120 days on selective medium. LBA4404 strain did not show transformation events.

Strain	Cocultivation (hours)	OD_600 nm_	Transformation events
AGL1	48	0.075	1.1 ± 0.2^e^
AGL1	48	0.15	4.4 ± 0.7^cd^
AGL1	48	0.3	7.7 ± 0.9^bc^
AGL1	72	0.075	2.1 ± 0.6^de^
AGL1	72	0.15	10.3 ± 1.2^ab^
AGL1	72	0.3	11.4 ± 1.6^a^
EHA105	48	0.075	0.0 ± 0.0^e^
EHA105	48	0.15	0.0 ± 0.0^e^
EHA105	48	0.3	0.7 ± 0.2^e^
EHA105	72	0.075	0.0 ± 0.0^e^
EHA105	72	0.15	0.7 ± 0.4^e^
EHA105	72	0.3	0.9 ± 0.3^e^

The data correspond to the mean values ± standard error in three independent experiments (*n* = 3). Different letters represent significant differences (Holm-Sidak, *α* = 0.05).
