# Supplementary material for: Stable Agrobacterium-Mediated Transformation of Maritime Pine Based on Kanamycin Selection
Source: ScientificWorldJournal. 2013 Nov 24;2013:681792. doi: 10.1155/2013/681792 (PMC3859213; doi:10.1155/2013/681792)

Online Resource 1

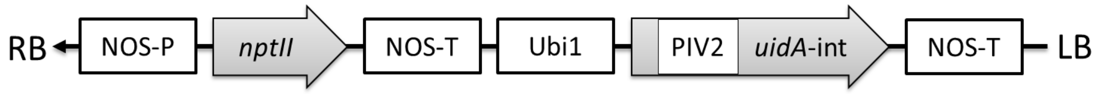

Online Resource 2

Online Resource 2 Primer sequences used for PCR amplification and Real Time PCR (qPCR).

Gene	Amplicon length	Sequence (5' - 3')
Pips-C61 (qPCR)	93 bp	ACACGCAGGACACAATAC<br>CTGCTGCTGGTGATTTTG
uidA (qPCR)	146 bp	GCAAGCGCACTTACAGGCGA<br>ACGGCGTGACATCGGCTTCAA
nptII	780 bp	GAGGCTATTCGGCTATGACTG<br>ATCGGGAGCGGCGATACCGTA
uidA	1199 bp	GGTGGGAAAGCGCGTTACAAG<br>GTTTACGCGTTGCTTCCGCCA
virG	200 bp	AAGGTGAGCCGTTGAAACAC<br>ATCTCAAGCCCATCTTCACG

Online Resource 3

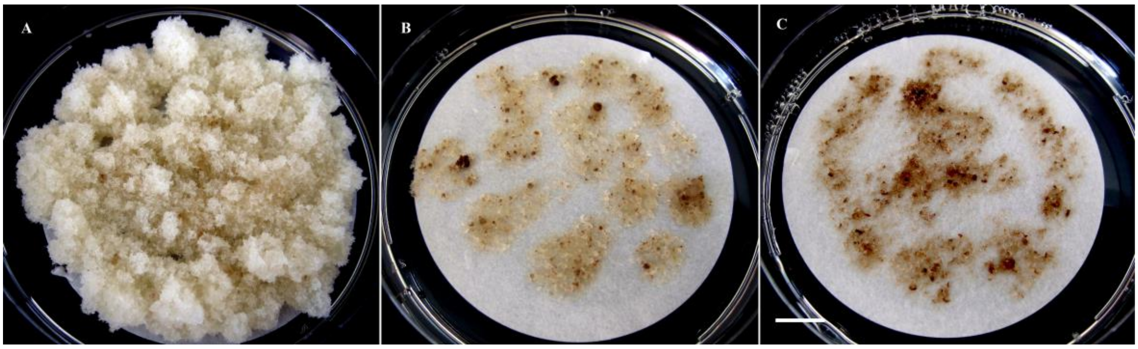

Online Resource 4

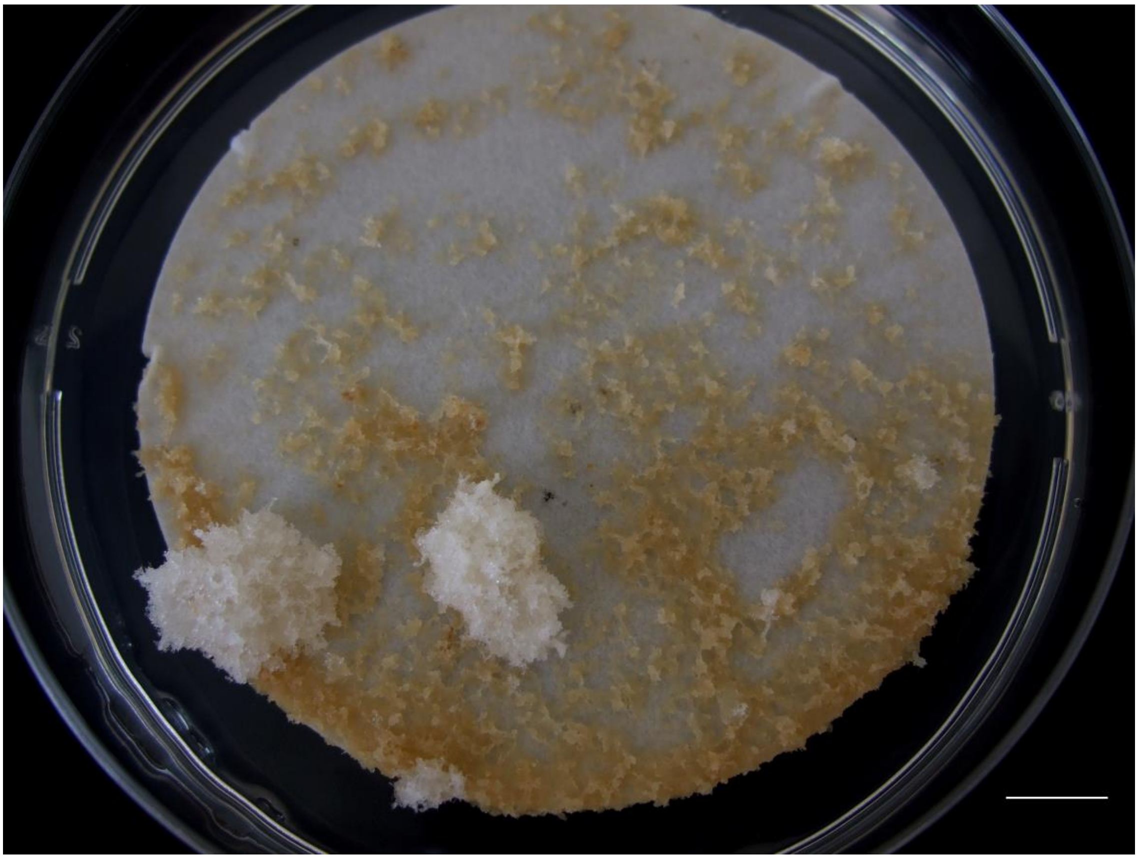

Online Resource 5

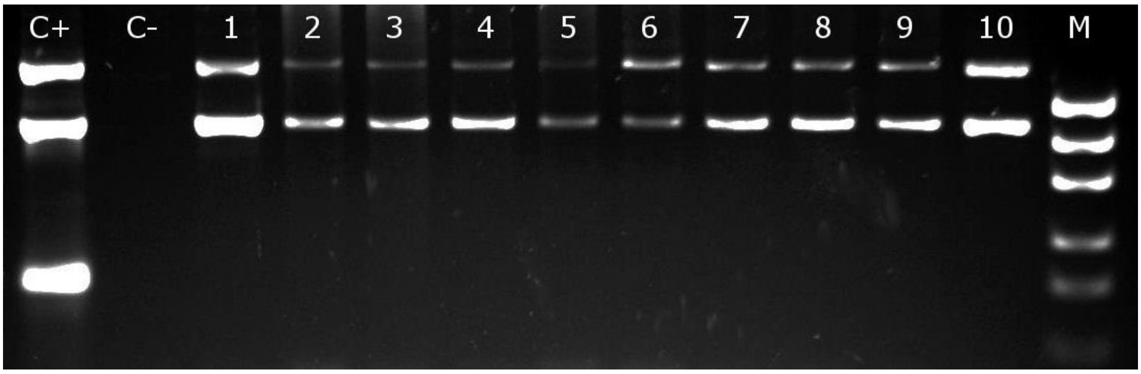

Online Resource 6

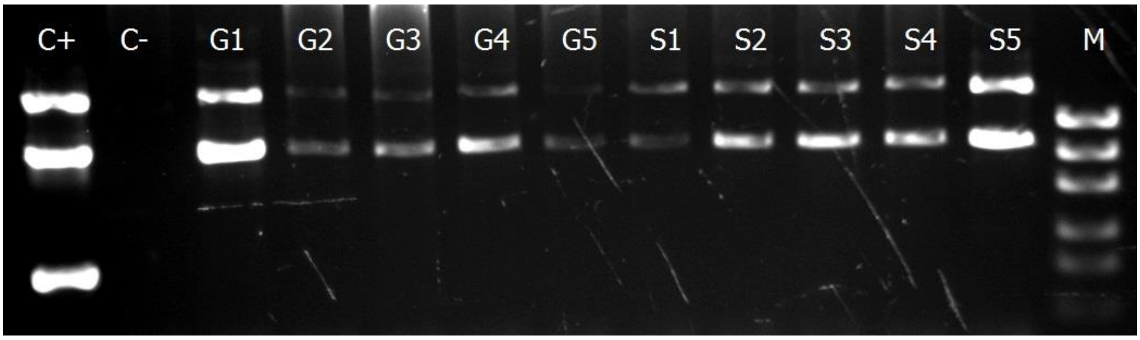

Supplement: Supplementary file 1 — Supplementary Data: Supplementary Data are available online. Online Resource 1: T-DNA structure of the binary vector pBINUbiGUSint. Online Resource 2: Primer sequences used for PCR amplification and Real Time PCR (qPCR). Online Resource 3: Appearance of the EM after 3 subcultures (6 weeks) on selective medium. Online Resource 4: Putatively transformed masses. Online Resource 5: PCR amplification of the genes nptII, uidA, and virG in 10 putative transgenic lines. Online Resource 6: PCR amplification of the genes nptII, uidA, and virG in 5 transgenic plants and 5 rooted shoots. [file 681792.f1.pdf]
